# Does Concurrent Cholestasis Alter the Prognostic Value of Preoperatively Elevated CA19-9 Serum Levels in Patients with Pancreatic Head Adenocarcinoma?

**DOI:** 10.1245/s10434-022-12460-w

**Published:** 2022-09-12

**Authors:** Friedrich Anger, Johan Friso Lock, Ingo Klein, Ingo Hartlapp, Armin Wiegering, Christoph-Thomas Germer, Volker Kunzmann, Stefan Löb

**Affiliations:** 1 Department of General, Visceral, Transplantation, Vascular and Paediatric Surgery, Julius Maximilians University Wuerzburg, Wuerzburg, Germany; 2 Department of Internal Medicine II, Julius Maximilians University Wuerzburg, Wuerzburg, Germany; 3 Comprehensive Cancer Centre Mainfranken, Julius Maximilians University Wuerzburg, Wuerzburg, Germany

## Abstract

**Background:**

Pancreatic adenocarcinoma (PDAC) patients with preoperative carbohydrate antigen 19-9 (CA19-9) serum levels higher than 500 U/ml are classified as biologically borderline resectable (BR-B). To date, the impact of cholestasis on preoperative CA19-9 serum levels in these patients has remained unquantified.

**Methods:**

Data on 3079 oncologic pancreatic resections due to PDAC that were prospectively acquired by the German Study, Documentation and Quality (StuDoQ) registry were analyzed in relation to preoperative CA19-9 and bilirubin serum values. Preoperative CA19-9 values were adjusted according to the results of a multivariable linear regression analysis of pathologic parameters, bilirubin, and CA19-9 values.

**Results:**

Of 1703 PDAC patients with tumor located in the pancreatic head, 420 (24.5 %) presented with a preoperative CA19-9 level higher than 500 U/ml. Although receiver operating characteristics (ROC) analysis failed to determine exact CA19-9 cut-off values for prognostic indicators (R and N status), the T, N, and G status; the UICC stage; and the number of simultaneous vein resections increased with the level of preoperative CA19-9, independently of concurrent cholestasis. After adjustment of preoperative CA19-9 values, 18.5 % of patients initially staged as BR-B showed CA19-9 values below 500 U/ml. However, the postoperative pathologic results for these patients did not change compared with the patients who had CA19-9 levels higher than 500 U/ml after bilirubin adjustment.

**Conclusions:**

In this multicenter dataset of PDAC patients, elevation of preoperative CA19-9 correlated with well-defined prognostic pathologic parameters. Bilirubin adjustment of CA19-9 is feasible but does not affect the prognostic value of CA19-9 in jaundiced patients.

**Supplementary Information:**

The online version contains supplementary material available at 10.1245/s10434-022-12460-w.

Pancreatic adenocarcinoma (PDAC) remains an aggressive gastrointestinal malignancy with a poor prognosis and currently is the fourth most common cause of cancer-related death in the Western world.^1^ Only 20 to 30% of patients with a diagnosis of PDAC present with anatomically resectable tumors.^2,3^ Surgical resection in combination with systemic chemotherapy offers the only option for long-term survival or even cure for patients with pancreatic cancer. However, modern multimodal treatment approaches still result in 5-year-survival rates no higher than 20 to 30%.^2^

Local recurrence rates of 77% attest to the aggressive tumor biology of PDAC. More than 50% recur at single distant sites within the first year after resection, suggesting the presence of systemic micrometastases at the time of diagnosis.^4,5^ Thus, the addition of more prognostic parameters to the preoperative staging algorithms would be helpful for identifying patients at high risk of early locoregional or systemic progression who may benefit from neoadjuvant systemic treatment rather than a surgery-first approach.^6^

Historically, upfront surgery followed by adjuvant therapy has been the standard of care for patients with primarily resectable pancreatic head cancer. In 2006, the National Comprehensive Cancer Network (NCCN) introduced criteria that classified PDAC as resectable (R), borderline resectable (BR), or unresectable (UR). The latter includes locally advanced disease (LA) or metastatic disease by means of locoregional tumor growth or distant metastases displayed in cross-sectional imaging.^7^

Accumulating evidence indicates that patients with BR- or LA-PDAC benefit from neoadjuvant multimodal therapy as findings have shown treatment failure of upfront surgery to be common.^8^ However, preoperative imaging-based staging is known to be unreliable in both the prediction of resectability and the detection of micrometastases that would preclude a curative approach.^9,10^ For that matter, the international consensus conference extended the definition of borderline resectability in 2017 by adding elevated preoperative serum levels of carbohydrate-antigen 19-9 (CA19-9) because studies have proven it to be the best available prognostic biomarker for PDAC patients.^11–14^ Consequently, the definition of borderline resectability has shifted away from exclusively anatomic measures, broadening to anatomic (BR-A) and biologic (BR-B) criteria.^15,16^

The prognostic importance of biologic borderline resectability (BR-B) in PDAC patients was recently shown in a European bicentric analysis. Patients with CA19-9 values higher than 500 U/ml showed not only locally advanced disease upon pathologic examination, but also an equal impairment of disease-free and overall survival compared with patients who had anatomically resectable PDAC.^17^

However, some scepticism has remained in daily clinical practice concerning preoperative CA19-9 levels as a biologic marker in PDAC patients. Patients with a lack of the Lewis antigen A (blood type antigen), which accounts for about 10% of the Caucasian population, will not secrete CA19-9. Furthermore, CA19-9 levels may be increased in cholestatic jaundice.^18^ The laboratory results for this subset of patients might fail to reflect the genuine CA19-9 level secreted by the tumor. Acute pancreatitis, which can accompany pancreatic carcinoma, might account for false-positive CA19-9 levels.^18^ Moreover, cut-off values are hard to define because receiver operating characteristics (ROC) analysis has yielded contrary results in small PDAC patient cohorts.^19,20^

In terms of defining resectability of pancreatic head cancer, a correlation between serum levels of CA19-9 and bilirubin was described, but with a weak correlation coefficient.^13^ In addition, preoperative hyperbilirubinemia was shown not to hamper the prediction of resectability in PDAC patients.^20^

Although CA19-9 is considered the gold standard of prognostic biomarkers in PDAC, the impact of jaundice on preoperative CA19-9 serum levels in PDAC patients has remained unquantified. The purpose of this German Study, Documentation and Quality (StuDoQ) pancreatic registry study was to evaluate whether concurrent cholestasis altered the prognostic value of preoperatively elevated serum CA19-9 levels in patients who underwent resection surgery for pancreatic head adenocarcinoma.

## Materials and Methods

### Data Source

Data were retrieved from the German StuDoQ registry, which was set up by the German Society for General and Visceral Surgery (DGAV) in 2010 to evaluate the quality of health care and risk factors for different types of surgery depending on the indicative disease. The StuDoQ|Pancreatic surgery registry (www.dgav.de/studoq; www.en.studoq.de) is a prospective registry that contains anonymized data of patients with pancreatic diseases treated in German hospitals.

Data from the participating clinics were included in a pseudonymized form and subjected to automatic plausibility controls. Validation by cross-checking with institutional medical controlling data is part of the annual certification process.

The following items available from StuDoQ|Pancreatic surgery were analyzed: age, gender, comorbidity (including American Association of Anesthesiologists [ASA] score^21^ and Charlson Comorbidity Index^22^), imminent preoperative laboratory results, postoperative histopathologic parameters (tumor location, histology, pathologic tumor-node-metastasis [pTNM] stage, resection margins), treatment details (surgical approach, venous resection, operation time, and blood loss), postoperative course (complications graded by Clavien-Dindo^23^), hospital length of stay, and in-hospital mortality.

Indication for performance of pancreaticoduodenectomy was based on interdisciplinary conferences in the individual center. The preoperative resectability classification of pancreatic tumors according to NCCN guidelines was not available.^15^ Tumor stage was classified according to the TNM classification (8th edition) of the American Joint Committee on Cancer/Union Internationale Contre le Cancer (AJCC/UICC-TNM).^24^ The patient’s R0 resection status was defined as no detectable tumor cells at the transection or circumferential margin (CRM) according to the currently valid definition of “CRM narrow.”^25^

### Study Population

The inclusion criteria from the StuDoQ|Pancreatic surgery registry (2014–2019) specified confirmed diagnosis of ductal adenocarcinoma of the pancreas (PDAC), pancreatic resection, and preoperative determination of CA19-9 serum levels. A dataset of 3079 patients was transferred from the registry and analyzed for further patient selection.

The exclusion criteria for further analysis ruled out non-pancreatic head tumor localization, evidence of any distant metastases, preoperative neoadjuvant therapy, incomplete pathologic results, performance status of 2 or higher according to the Eastern Cooperative Oncology Group (ECOG), and missing data concerning surgery and preoperative serum bilirubin. According to previous studies, non-secretors of CA19-9 were defined as those with a serum level lower than 2 U/ml and excluded from further analysis.^14^

This analysis was in accordance with the ethical standards of the German Society for General and Visceral Surgery (DGAV) StuDoQ registry and with the 1964 Helsinki declaration and its later amendments. Informed consent for data acquisition as well as data storage and safety by the StuDoQ registry was obtained from all the patients included in this study.

### Definitions

**Table:** 

BR-B	Borderline resectability due to tumor biology based on preoperative serum CA19-9 levels higher than 500 U/ml
Non-BR-B	Preoperative serum CA19-9-levels lower than 500 U/ml
BR-B^Corr^	Preoperative serum CA19-9 levels after adjustment by preoperative serum bilirubin levels higher than 500 U/ml
Non-BR-B^Corr^	Preoperative serum CA19-9 levels after adjustment by preoperative serum bilirubin levels lower than 500 U/ml
Correction to non-BR-B	Progression to a CA19-9 level below the threshold of 500 U/ml due to adjustment by preoperative bilirubin levels
CA19-9^corr^	CA19-9 values adjusted by preoperative bilirubin levels

### Statistical Analyses and Outcome Measures

All statistical analyses were performed using IBM SPSS Statistics 26 (International Business Machines Corporation, Armonk, NY, USA). Descriptive data are reported as medians with ranges or as totals with percentages (unless otherwise indicated). Univariate analysis was performed using chi-square, Fisher’s exact, or one-way analysis of variance (ANOVA) test according to the data scale and distribution.

The prognostic value of CA19-9 predicting positive N or R stage, was assessed using ROC curve analysis. The Spearman correlation coefficient was calculated to assess the effect of hyperbilirubinemia on CA19-9 serum levels.

A backward multivariable linear regression analysis was performed to display preoperative CA19-9 as a function of relevant tumor characteristics (TNM) and patient characteristics associated with the tumor (age, gender, weight loss, biliodigestive stent placement) and hyperbilirubinemia. The biologic variables CA19-9 and bilirubin were logarithmized. The resulting model was applied for calculation of an individually corrected CA19-9 level (CA19-9^Corr^) as follows:

$$_{{{\text{Log}}}} {\text{CA19}} - {9 } = { 1}.{1 } + \, 0.{24 } \times_{{{\text{log}}}}{\text{Bili }} + \, 0.{16 } \times {\text{ pT }} + \, 0.0{6 } \times {\text{ pN }} + \, 0.{5 } \times {\text{Age }}\left( {{\text{Decade}}} \right)$$

$${\text{CA19}} - {9 } = { 13}.0 \, \times { 1}.{73 } \times_{{{\text{log}}}}{\text{Bili }} \times { 1}.{43 } \times {\text{ pT }} \times { 1}.{16 } \times {\text{ pN }} \times { 1}.{13 } \times {\text{Age }}\left( {{\text{Decade}}} \right)$$

At bilirubin levels lower than 3.6 mg/dl, no correction was performed because this had resulted in an increase in correction of CA19-9 levels. Thus, decreasing correction was performed only for patients with hyperbilirubinemia. Two-sided *p* values lower than 0.05 were considered statistically significant.

Factors independently associated with CA19-9 were _Log_Bilirubin, pathologic T and N status, age (decade), and biliary stent placement (Table S1).

## Results

### Patient Characteristics

Altogether, 1703 PDAC patients registered in StuDoQ met the inclusion criteria of this study (Fig. S1). The cohort consisted of 914 male and 692 female patients with a median age of 70 years (range, 31–99 years) and a mean preoperative body mass index (BMI) of 24.9 kg/m^2^ (range, 15.5–57.1 kg/m^2^). The most common ASA scores were 2 and 3 (94.3 %), whereas the Charlson Comorbidity Index (CCI) was moderate in most cases (51 %) accordingly. The median preoperative serum bilirubin level was 2 mg/dl (range, 1.1–47.9 mg/dl).

Of the 1703 patients, 723 (42.5 %) underwent preoperative bile drainage before CA19-9 measurement. The median CA19-9 level was 137.7 U/ml (range, 2–28.768 U/ml). According to the International Consensus Criteria ,420 patients (24.7 %) were classified as borderline resectable (BR-B) in terms of tumor biology, with a preoperative serum CA19-9 level higher than 500 U/ml. The patients with a preoperative serum CA19-9 level lower than 500 U/ml were categorized as non-BR-B.

The BR-B patients were significantly older (71 vs 70 years; *p* = 0.010) and presented with significantly higher preoperative levels of C-reactive protein (CRP 7.7 mg/dl [range, 0.1–346 mg/dl] vs 5.2 mg/dl [range, 0.1–214 mg/dl]; *p* = 0.001), bilirubin (4.5 mg/dl [range, 1.1–45.4] vs. 1.6 mg/dl [range, 1.1–47.9]; *p* < 0.001) and yGT (452 U/l [range, 0.2–4.252] vs. 264 U/l [range, 0.2–4.576]; *p* = 0.004) than the non-BR-B patients. Although significantly more patients presented with an icterus in the BR-B group (49.6 % vs 42.7 %; *p* = 0.013), biliodigestive stent placement did not differ between the groups (44.2 % vs 41.9 %; *p* = 0.426). The BR-B patient group had a significantly higher number of simultaneous venous resections (25.5 % vs 18.0 %; *p* = 0.001) and intraoperative blood transfusions (15.5 % vs 9.9 %; *p* = 0.002). The patient characteristics are listed in Table 1.

**Table 1:** Patient characteristics, preoperative laboratory results, surgery, and postoperative course

	Total (*n* = 1703) *n* (%)	Non-BR-B (*n* = 1283) *n* (%)	BR-B (*n* = 420) *n* (%)	*p* value
*Patient characteristics*				
Median age: years (range)	70 (31–99)	70 (31–99)	71 (44–91)	**0.010**
Male sex male	915 (53.7)	692 (53.9)	223 (53.1)	0.764
Median BMI: kg/m^2^ (range)	24.9 (15.5–57.1)	24.9 (15.5–57.1)	25.0 (15.6–48.3)	0.175
ASA status >2	880 (51.7)	657 (51.2)	223 (53.1)	0.909
CCI				0.103
Minor (1–4)	588 (34.5)	456 (36.3)	132 (32.0)	
Moderate (5–6)	868 (51.0)	650 (51.8)	218 (52.8)	
Major (≥7)	212 (12.4)	149 (11.9)	63 (15.3)	
Jaundice	756 (44.4)	547 (42.7)	209 (49.6)	**0.013**
Stenting DHC	723 (42.5)	537 (41.9)	186 (44.2)	0.426
*Preoperative laboratory results*				
Median CRP: mg/l (range)	5.6 (0.1–346.0)	5.2 (0.1–214.0)	7.7 (0.1–346)	**0.001**
Median bilirubin: mg/dl (range)	2.0 (1.1–47.9)	1.6 (1.1–47.9)	4.5 (1.1–45.4)	**< 0.001**
Median gamma-glutamyl-transferase U/l (range)	301 (0.2–4576)	264 (0.2–4576)	452 (0.2–4252)	**0.004**
Median lipase: U/l (range)	72.0 (0.1–5534.0)	71.0 (0.1–5534.0)	75.0 (0.1–5273)	0.119
Median CEA: ng/ml (range)	3.0 (0.1–2406.0)	2.7 (0.1–2406)	4.2 (0.5–249.0)	0.663
Median CA19-9: U/ml (range)	137.7 (2.0–28768)	74.8 (2.0–499)	1200.0 (500–28768)	**< 0.001**
*Surgery and postoperative course*				
Surgical procedure				0.269
PPPD	1016 (59.7)	764 (59.5)	252 (60.0)	
PRPD	464 (27.2)	342 (26.7)	122 (29.0)	
PD	223 (13.1)	177 (13.8)	46 (11.0)	
Median operation time: min (range)	330 (55–735)	330 (78–722)	335 (55–735)	0.510
Resection PV/SMV	338 (19.8)	231 (18.0)	107 (25.5)	**0.001**
Intraoperative transfusion of RBC	190 (11.2)	126 (9.9)	64 (15.5)	**0.002**
Median LOS: days (range)	19.5 (18.9–20.1)	19.7 (19.0–20.4)	19.2 (18.1–20.3)	0.451
	16 (0–116)	16 (0–116)	16 (0–87)	
Clavien-Dindo ≥3b	295 (17.3)	227 (17.7)	68 (16.2)	0.480
In-house mortality	76 (4.5)	56 (4.4)	20 (4.8)	0.732

*P* values considered statistically significant are presented in bold

Non-BR-B, preoperative serum CA19-9 <500 U/ml; BR-B, preoperative serum CA19-9 >500 U/ml (definition according ref 11); BMI, body mass index; ASA, American Society of Anesthesiologists; CCI, Charlson Comorbidity Index; DHC, ductus hepatocholedochus; CRP, C-reactive protein; CEA, carcinoembryonic antigen; CA19-9, carbohydrate antigen 19-9; PPPD, pylorus-preserving pancreaticoduodenectomy; PRPD, pylorus-resecting partial pancreaticoduodenectomy; PD, total pancreaticoduodenectomy; PV, portal vein; SMV, superior mesenteric vein; RBC, red blood cells; LOS, hospital length of stay.

### Upfront Surgery for Biologically Resectable PDAC Results in Higher Rates of Positive Resection Margins and Advanced Tumor Stages

According to the TNM system (8th edition of TNM) introduced by the AJCC/UICC-TNM, most patients were classified into T3 (53.7 %) and N1 (51.4 %) categories. Tumor (T) and nodal (N) stages were significantly higher for the BR-B patients (*p* < 0.001), with an N2 rate of 24.7 %. Accordingly, the BR-B patients presented with significantly higher UICC stages than the non-BR-B patients (UICC 3: 28.6 % vs 20.0 %; *p* < 0.001).

The patients showed marked intergroup differences in terms of positive resection margins (R1). Pathologic examination of the pancreatic head specimens showed that positive resection margins occurred significantly more often among the patients staged as BR-B (27.2 %) than among the non-BR-B patients (21.1 %) (*p* = 0.012).

Because the definition of R1 status changed during the study period, a subgroup analysis of the patients whose specimens had examination of a circumferential resection margin (CRM) was performed. In the subgroup analysis, R1 status increased to 42.2 % among the patients staged as BR-B compared with 29.1% among the patients staged as non-BR-B (*p* = 0.003). The patients classified as BR-B showed significantly worse tumor grading than those assigned to the non-BR-B group (*p* = 0.045). No differences with regard to lymphatic vessel (L), microvascular (V), or perineural (Pn) infiltration rates were detected. Detailed pathologic information is outlined in Table 2.

**Table 2:** Pathologic parameters by definition of biologic borderline resectability

	Non-BR-B (*n* = 1283) *n* (%)	BR-B (*n* = 420) *n* (%)	*p* value
T status			**<0.001**
1	109 (8.5)	11 (2.6)	
2	481 (37.5)	138 (32.8)	
3	660 (51.1)	254 (60.3)	
4	32 (2.5)	18 (4.3)	
N status			**<0.001**
1	643 (50.2)	233 (55.3)	
2	232 (18.1)	104 (24.7)	
R status			**0.012**
Positive	268 (21.1)	113 (27.2)	
R status (CRM)^a^			**0.003**
R0 wide	268 (45.3)	57 (33.5)	
R0 narrow	151 (25.5)	41 (24.1)	
R1	172 (29.1)	72 (42.4)	
L status			0.082
1	622 (48.5)	225 (53.4)	
V status			0.225
1	277 (21.6)	103 (24.5)	
Pn status			0.074
1	968 (75.5)	336 (79.8)	
G status			**0.045**
1	48 (3.8)	11 (2.6)	
2	723 (56.8)	212 (50.4)	
3	497 (39.0)	195 (46.3)	
4	6 (0.5)	3 (0.7)	
UICC stage			**<0.001**
1	225 (17.5)	35 (8.3)	
2	802 (62.5)	265 (63.1)	
3	256 (20.0)	120 (28.6)	

*P* values considered statistically significant are presented in bold

*Non-BR-B * Peoperative serum CA19-9 <500 U/ml; *BR-B* Preoperative serum CA19-9 >500 U/ml (definition according ref 11); *CRM* Circumferential margin; *UICC* Union Internationale Contre le Cancer

^a^Patients with CRM examined and discrimination between R0 wide and narrow

### Preoperative CA19-9 is a Weak Predictor of Postoperative Pathologic R and N Status

To evaluate the predictive value of CA19-9 for the most relevant prognostic tumor characteristics in terms of local invasiveness, ROC analysis of CA19-9 as a function of N and R status was performed. The results showed CA19-9 to be a weak predictor of R1 (area under the curve [AUC], 0.547; *p* = 0.005), N2 (AUC, 0.566; *p* < 0.001), and positive N (AUC, 0.601; *p* < 0.001) status, failing to provide an adequate cut-off value for preoperative CA19-9 serum levels (Fig. S2).

### Hyperbilirubinemia Does not have an Impact on the Prognostic Value of CA19-9 in PDAC Patients

**Fig. 1:**
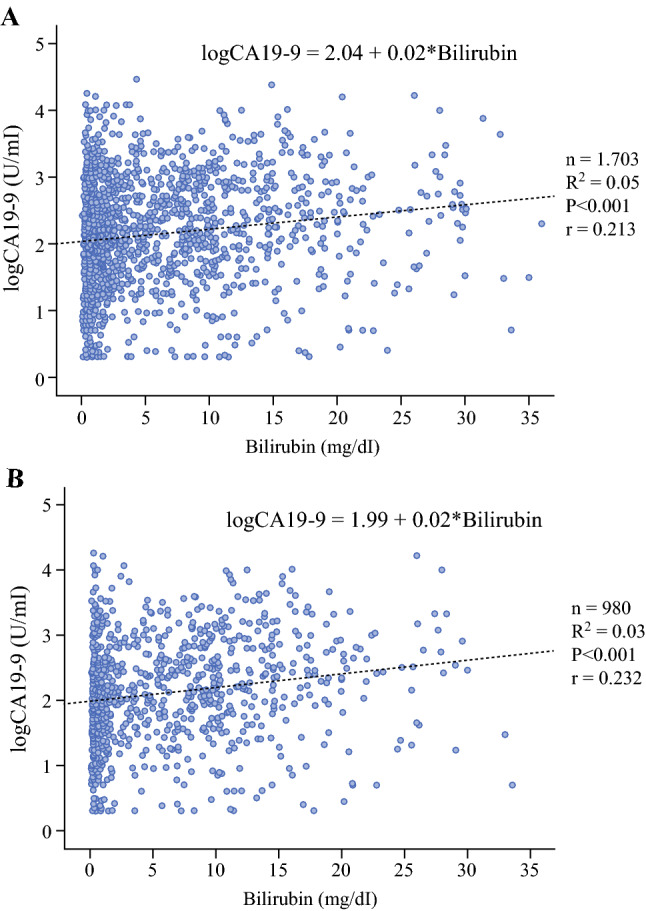
Correlation of preoperative serum CA19-9 and bilirubin levels in **A** all the patients and **B** the patients who received biliary stent placement before CA19-9 measurement. CA19-9, carbohydrate antigen 19-9

First, we performed a correlation analysis of preoperative CA19-9 and bilirubin in patients’ serum. The analysis showed a statistically significant correlation between hyperbilirubinemia and CA19-9 levels (<0.001), but the correlation coefficients of 0.213 for all the patients (Fig. 1A) and 0.232 for the patients who received stent placement before CA19-9 measurement (Fig. 1B) attested to a minor interaction.

Second, the CA19-9 values for all the patients were corrected by bilirubin values according to the results of the linear regression analysis. This moved 78 patients (18.5 %) below the CA19-9 cut-off of 500 U/ml, and they therefore no longer met the biologic criteria of borderline resectability (correction to non-BR-B; Table 3).

**Table 3:** Impact of CA19-9 correction by bilirubin on BR-B classification and postoperative pathologic parameters

	BR-B (*n* = 420) *n* (%)	Correction to Non-BR-B (*n* = 78) *n* (%)	BR-B^corr^ (*n* = 343) *n* (%)	*p* value
Bilirubin: mg/dl (range)	4.5 (1.1–45.4)	12.6 (1.8–39.8)	2.8 (1.1.–45.4)	**< 0.001**
T status				0.981
1	11 (2.6)	2 (2.6)	9 (2.6)	
2	138 (32.8)	25 (32.1)	113 (32.9)	
3	254 (60.3)	47 (60.3)	207 (60.3)	
4	18 (4.3)	4 (5.1)	14 (4.1)	
N status				0.687
1	233 (55.3)	46 (59.0)	187 (54.5)	
2	104 (24.7)	19 (24.4)	85 (24.8)	
R status Positive	113 (27.2)	20 (26.3)	93 (27.4)	1.000
R status (CRM)^a^	(*n* = 170)	(*n* = 35)	(*n* = 135)	0.528
R0 wide	57 (33.5)	14 (40.0)	43 (31.9)	
R0 narrow	41 (24.1)	9 (25.7)	32 (23.7)	
R1	72 (42.4)	12 (34.3)	60 (44.4)	
L status				**0.033**
1	225 (53.4)	33 (42.3)	192 (56.0)	
V status				0.884
1	103 (24.5)	18 (23.1)	85 (24.8)	
Pn status				0.755
1	336 (79.8)	61 (78.2)	275 (80.2)	
G status				0.059
1	11 (2.6)	2 (2.6)	9 (2.6)	
2	212 (50.4)	32 (41.0)	180 (52.5)	
3	195 (46.3)	42 (53.8)	153 (44.6)	
4	3 (0.7)	2 (2.6)	1 (0.3)	

BR-B, preoperative serum CA19-9 >500 U/ml (definition according ref 11); Non-BR-B, preoperative serum CA19-9 <500 U/ml; CRM, circumferential margin

^a^Patients with CRM examined and discrimination between R0 wide and narrow

To detect changes in the prognostic value of bilirubin-corrected CA19-9 (CA19-9^corr^) values in terms of tumor characteristics, the cohort of patients with a CA19-9 correction to non-BR-B status was compared with the cohort of patients still categorized as BR-B after CA19-9 correction (BR-B^corr^). This resulted in no detectable change in T, N, or R status, nor in V, Pn, or G status. Relevant changes occurred only in the L status, with a significantly lower L1 among the patients who had a CA19-9 correction below 500 U/ml (42.3 % vs 56.0 %; *p* = 0.033). Altogether, it can be stated that concurrent hyperbilirubinemia does not alter the prognostic value of CA19-9, even in jaundiced patients.

### Bilirubin Correction Does not Change the Predictive Value of Preoperative CA19-9 for Postoperative Pathologic R and N Status

To re-evaluate the predictive value of CA19-9 for the most relevant prognostic tumor characteristics after correction by concurrent hyperbilirubinemia, ROC analysis of CA19-9 as a function of N and R status was repeated. However, the predictive value of CA19-9^Corr^ for R1 (AUC, 0.548; *p* = 0.004), N2 (AUC, 0.565; *p* < 0.001), and positive N (AUC, 0.595; *p* < 0.001) status did not change compared with that of non-corrected CA19-9. Thus, appropriate cut-off values for preoperative CA19-9^Corr^ serum levels could not be identified (Fig. S3).

### Preoperative CA19-9 Serum Levels are Significantly Associated with Venous Resection Rate and Postoperative Pathologic Parameters

The more that CA19-9 serum levels were preoperatively increased, the more simultaneous vein resections during pancreaticoduodenectomy were performed and the more patients presented with larger tumors, more lymph node metastases, poorer tumor differentiation, higher tumor stages, and ultimately positive resection margins (Table 4). This association of preoperative CA19-9 serum levels with venous resection rates and postoperative prognostic pathologic parameters remained unchanged in a subgroup analysis of the patients with concurrent hyperbilirubinemia (bilirubin ≥3.78 mg/dl; Table 5). Thus, the prognostic value of CA19-9 was not hampered by concurrent hyperbilirubinemia.

**Table 4:** Venous resection rates and pathologic results according to clustered preoperative carbohydrate antigen 19-9 (CA19-9) levels

CA 19-9	*n*	Bilirubin (mg/dl)	Vein resection *n* (%)	≥T3 *n* (%)	≥ N1 *n* (%)	= N2 *n* (%)	≥ R1 *n* (%)	= R0 wide^a^ *n* (%)	≥ G3 *n* (%)	UICC ≥ 3 *n* (%)
<37 U/ml	414	1.2	59 (14.3)	214 (51.7)	255 (61.6)	59 (14.3)	89 (21.5)	106 (32.2)	156 (37.9)	69 (16.4)
37 to <250 U/ml	643	1.7	124 (19.3)	347 (54.0)	436 (67.8)	122 (19.0)	121 (18.8)	163 (32.5)	243 (38.0)	133 (20.7)
250 to <500 U/ml	225	2.7	48 (21.3)	131 (58.2)	184 (81.8)	51 (22.7)	58 (25.8)	51 (26.7)	104 (46.8)	54 (24.0)
500 to <1000 U/ml	174	5.5	44 (25.3)	112 (64.4)	140 (80.5)	42 (24.1)	53 (30.5)	36 (26.5)	86 (49.4)	48 (27.6)
≥1000 U/ml	247	4.3	63 (25.5)	160 (64.8)	197 (79.8)	62 (25.1)	60 (24.3)	35 (17.7)	112 (45.3)	72 (29.1)
*p* value	–	<0.001	< 0.001	< 0.001	< 0.001	< 0.001	0.018	< 0.001	0.002	< 0.001

*UICC* Union Internationale Contre le Cancer; *CRM* circumferential margin

^a^Patients with CRM examined and discrimination between R0 wide and narrow

**Table 5:** Venous resection rates and pathologic results according to clustered preoperative carbohydrate antigen 19-9 (CA19-9) levels in patients with simultaneous cholestasis (bilirubin ≥3.78 mg/dl)

CA 19-9	*n*	Bilirubin (mg/dl)	Vein resection *n* (%)	≥ T3 *n* (%)	≥ N1 *n* (%)	= N2 *n* (%)	≥ R1 *n* (%)	= R0 wide^a^ *n* (%)	≥ G3 *n* (%)	UICC ≥3
<37 U/ml	120	10.2	13 (10.8)	72 (60.0)	71 (59.2)	16 (13.3)	24 (20.0)	39 (32.5)	44 (37.0)	17 (14.2)
37 to <250 U/ml	234	9.6	46 (19.7)	133 (56.8)	167 (71.4)	48 (20.5)	46 (19.7)	60 (25.6)	90 (38.5)	52 (22.2)
250 to <500 U/ml	99	9.8	24 (24.2)	59 (59.6)	80 (80.8)	25 (25.3)	25 (25.5)	23 (23.5)	50 (51.5)	27 (27.3)
500 to <1000 U/ml	97	11.1	25 (25.8)	64 (66.0)	71 (73.2)	22 (22.7)	27 (28.4)	22 (23.2)	52 (53.6)	26 (26.8)
≥1000 U/ml	133	10.5	30 (22.6)	91 (61.3)	104 (78.2)	34 (25.6)	35 (26.7)	20 (15.3)	62 (46.6)	40 (30.1)
*p* Value	–	0.237	0.015	0.037	< 0.001	0.024	0.049	0.001	0.011	0.003

*UICC* Union Internationale Contre le Cancer; *CRM* circumferential margin

^a^Patients with CRM examined and discrimination between R0 wide and narrow

## Discussion

The prognosis of patients with PDAC remains dismal. Early local recurrence or distant metastases account for 5 year survival rates of only 20 to 30 % for patients after curative resection and modern adjuvant chemotherapy regimens.^2,26^ Biologic markers such as CA19-9 have been intensively investigated to identify patients with a higher risk of pre-existing micrometastases who might benefit from neoadjuvant therapeutic strategies. However, the specificity of preoperatively elevated CA19-9 levels remains controversially discussed, especially in the case of coexisting cholestasis.

This is the first study of this issue based on prospectively gathered, validated multicenter registry data using unitarily compiled preoperative laboratory results in a well-defined patient collective with resected pancreatic ductal adenocarcinoma of the pancreatic head. The results confirm an association of elevated preoperative serum CA19-9 with locally advanced disease in patients with resected PDAC. Remarkably, the extent of local disease (i.e., tumor stage, venous resection rate, nodal positivity, and positive resection margins) increases with preoperative CA19-9 serum levels. A correlation of preoperative CA19-9 serum and bilirubin levels has been shown in the past,^13,27^ although the correlation coefficients were low. Moreover, the clinical impact of this statistical result has remained unclear.

The current study presented a novel approach of CA19-9 correction in case of concurrent hyperbilirubinemia using a linear regression model. Most interestingly, the correction of preoperative CA19-9 by applying this new algorithm does not have an impact on its prognostic value in defining locally advanced disease.

In this study, 25 % of all the patients presented with a preoperative CA19-9 serum level that equalled or exceeded 500 U/ml and were consequently staged as biologically borderline resectable (BR-B) according to previously published data.^28,29^ The statistically significant difference in age between the patients staged BR-B and the non-BR-B patients remains without clinical significance, with a numeric difference of 1 year in the median and equal ranges. Interestingly, the BR-B patients presented with significantly higher CRP levels, suggesting a stronger tumor-associated inflammation in the BR-B patients. Tumor-associated inflammation is associated with prognosis because it is documented by the Glasgow Prognostic Score that combines serum CRP and albumin levels and stratifies prognosis in various cancers including PDAC.^30,31^ The combination of biologic and inflammatory tumor markers in PDAC was recently used to create a pretreatment score to stratify the survival of PDAC patients more precisely.^32^

Since the acquisition of preoperative serum CA19-9 levels that equal or exceed 500 U/ml in the definition of borderline resectability of PDAC patients during the 20th meeting of the International Association of Pancreatology,^11^ potential confounders of preoperative CA19-9 serum levels have regained the interest of oncologists and surgeons.^18,33^ The most relevant is hyperbilirubinemia, a frequent condition among patients with PDAC of the pancreatic head.^12,34,35^ This raises the question whether patients with concurrent cholestasis might present with false-positive CA19-9 serum levels.

In the current study, the patients classified as BR-B presented significantly more often with jaundice as well as higher levels of bilirubin and gamma-glutamyl-transferase in their imminent preoperative laboratory results. These indicate a greater extent of cholestatic conditions among these patients, raising the question whether elevated serum CA19-9 represents biliary stress or tumor growth. However, in cases of morphologically highly suspected or histologically proven pancreatic cancer, elevated CA19-9 serum levels have been shown to have a relevant prognostic value^13,17,18^ without correction for hyperbilirubinemia in jaundiced patients.

A look at the postoperative pathologic parameters of BR-B patients in this study showed significantly higher T, N, and ultimately UICC stages. Consequently, more simultaneous venous resections needed to be performed, triggering more intraoperative blood transfusions. It can be stated that a preoperative CA19-9 serum level of 500 U/ml or higher is associated with advanced local tumor growth and nodal positivity in up to 80% of these patients as well as a higher rate of extended surgical resections that result in a significantly higher rate of positive resection margins, thereby drastically altering patients’ prognosis, as previously published.^13,17,36^

It is well known that an elevated preoperative CA19-9 serum level is an independent predictor for nodal positivity,^14^ but it also predicts extended lymphatic metastasis.^17,37^ Nevertheless, ROC analysis did not provide acceptable sensitivity or specificity of preoperative CA19-9 values regarding the prediction of nodal or margin status despite the large patient cohort.

Several previous single-center studies failed to define adequate cut-off values for preoperative CA19-9 for predicting nodal positivity or margin status in PDAC patients.^19,38^ Therefore, the proposed CA19-9 cut-off level of 500 U/ml to define biologic borderline resectability as stated in the consensus conference was used for further analysis.

As a readily available marker, CA19-9 has failed in several studies to demonstrate reliable discrimination between benign and malignant bilio-pancreatic lesions.^33,39^ The studies all report a higher level of CA19-9 in case of malignancies and less decrease after biliary drainage in case of concurrent cholestasis, but no adequate cut-off values for discrimination of malignant lesions. Especially hyperbilirubinemia was associated with a further deterioration in specificity,^39^ raising the question whether the prognostic value of preoperative CA19-9 for PDAC patients might be biased the same way.

Although a correlation of preoperative CA19-9 and bilirubin levels in PDAC patients scheduled for resection surgery was demonstrated by Hartwig et al.^13^ in 2013, the current study is the first large-scale registry analysis to present context for the impact of cholestasis on the prognostic value of preoperative CA19-9. The basic concept relies on a multivariable linear regression analysis that showed an independent association of CA19-9, bilirubin with pathologic parameters. On this basis, a confounding tumor characteristic could be extracted from the correlation of bilirubin and CA19-9, thereby allowing for correction of CA19-9 by bilirubin.

After correction of CA19-9 by concurrent hyperbilirubinemia, only 18 % of the patients initially staged as BR-B moved to the non-BR-B patient group (correction with non-BR-B). However, these patients did not differ from the remaining BR-B^corr^ patients in terms of pathologic parameters and still presented with locally advanced tumors. The rate of lymphovascular tumor invasion is merely less frequently observed in patients corrected to non-BR-B, without a reasonable explanation in the underlying dataset. In summary, our results show for the first time that concurrent cholestasis does not influence the individual prognostic value of preoperative serum CA19-9 levels in patients with resectable PDAC of the pancreatic head.

This study had some limitations that need to be addressed. First, the registry does not provide data on the long-term outcome for patients. Thus, no statement about the impact of concurrent cholestasis on the prognostic value of CA19-9 for the overall or disease-free survival of PDAC patients can be provided. In this context, we note that this relationship has been extensively studied and clearly demonstrated in multiple former studies.^13,14,17^

Second, the registry provides data only about preoperatively collected laboratory results. Therefore, kinetics in bilirubin levels after ductus hepatocholedochus (DHC) stent placement cannot be provided.

Third, this registry analysis was prone to selection bias because patients with missing data had been excluded or were not retrieved from the registry at first.

On the other hand, the use of prospectively acquired and continuously updated clinical data is a significant strength of this study. Hospitals providing data to the registry typically seek certification by the German Society of General and Visceral Surgery (DGAV). Consequently, these data represent outcomes of patients treated in hospitals with a certified high standard of care. For example, the mortality rate after pancreatic head resections found in this study is considerably lower than in analyses based on nationwide data from German hospitals.^40^

In line with previous studies, this study clearly demonstrated that together with a rise in preoperative CA19-9 serum levels, a statistically significant increase in the probability of locally advanced tumor growth occurs for patients with resectable PDAC. This association, reflecting the actual prognostic value of preoperative elevated CA19-9 serum levels in PDAC patients, was not biased by concurrent cholestasis in this study. Due to the repetitively shown correlation of CA19-9 and bilirubinemia, there has been an ongoing debate on the impact that cholestasis has on the prognostic value of CA19-9 for PDAC patients. The results of this study confirm a correlation between CA19-9 and bilirubinemia, but indicate for the first time that correction of CA19-9 in case of hyperbilirubinemia does not alter its prognostic value. An individual interpretation of elevated preoperative CA19-9 serum levels in case of simultaneous cholestasis in patients with histologically proven or morphologically suspected PDAC might not be required. This study presented relevant results regarding the interpretation of preoperative CA19-9 serum levels in PDAC patients during clinical practice and in interdisciplinary conferences.

## Supplementary Information

Below is the link to the electronic supplementary material.

Supplementary file1 (DOCX 19 kb)

Supplementary file2 (DOCX 4593 kb)

## Data Availability

The data that support the findings of this study are available from the DGAV StuDoQ registry, but restrictions apply to the availability of these data, which were used under license (ID StuDoQ-2019-0010) for the current study, and therefore are not publicly available. Data are however available from the authors upon reasonable request and with permission of the DGAV StuDoQ registry.
