# Supplementary material for: Does Concurrent Cholestasis Alter the Prognostic Value of Preoperatively Elevated CA19-9 Serum Levels in Patients with Pancreatic Head Adenocarcinoma?
Source: Ann Surg Oncol. 2022 Sep 12;29(13):8523–33. doi: 10.1245/s10434-022-12460-w (PMC9640457; doi:10.1245/s10434-022-12460-w)
Supplement: Supplementary file 1 — Supplementary file1 (DOCX 19 kb) [file 10434_2022_12460_MOESM1_ESM.docx]

Supplementary Table 1:

| Factor | B (95% CI) | Beta | P-value |
| --- | --- | --- | --- |
| Constant (_Log_CA19-9) | 0.769 (0.362; 1.176) |  | **<0.001** |
| LogBilirubin | 0.312 (0.230; 0.394) | 0.178 | **<0.001** |
| T-Status | 0.159 (0.104; 0.215) | 0.134 | **<0.001** |
| N-Status | 0.063 (0.037; 0.089) | 0.111 | **<0.001** |
| G-Status | 0.58 (-0.008; 0.124) | 0.041 | 0.085 |
| L-Status | 0.025 (-0.055; 0.105) | 0.016 | 0.533 |
| V-Status | 0.008 (-0.086; 0.102) | 0.004 | 0.866 |
| Pn-Status | 0.015 (-0.075; 0.105) | 0.008 | 0.748 |
| Stent DHC | 0.077 (0.002; 0.152) | 0.048 | **0.045** |
| Gender | 0.007 (-0.068; 0.082) | 0.004 | 0.854 |
| Preoperative weight loss >10% within 6 months | -0.022 (0.103; 0.060) | -0.012 | 0.602 |
| Age (decade) | 0.056 (0.020; 0.092) | 0.072 | **0.002** |
