# Supplementary material for: Does Concurrent Cholestasis Alter the Prognostic Value of Preoperatively Elevated CA19-9 Serum Levels in Patients with Pancreatic Head Adenocarcinoma?
Source: Ann Surg Oncol. 2022 Sep 12;29(13):8523–33. doi: 10.1245/s10434-022-12460-w (PMC9640457; doi:10.1245/s10434-022-12460-w)
Supplement: Supplementary file 2 — Supplementary file2 (DOCX 4593 kb) [file 10434_2022_12460_MOESM2_ESM.docx]

**Supplementary Figure 1**

**
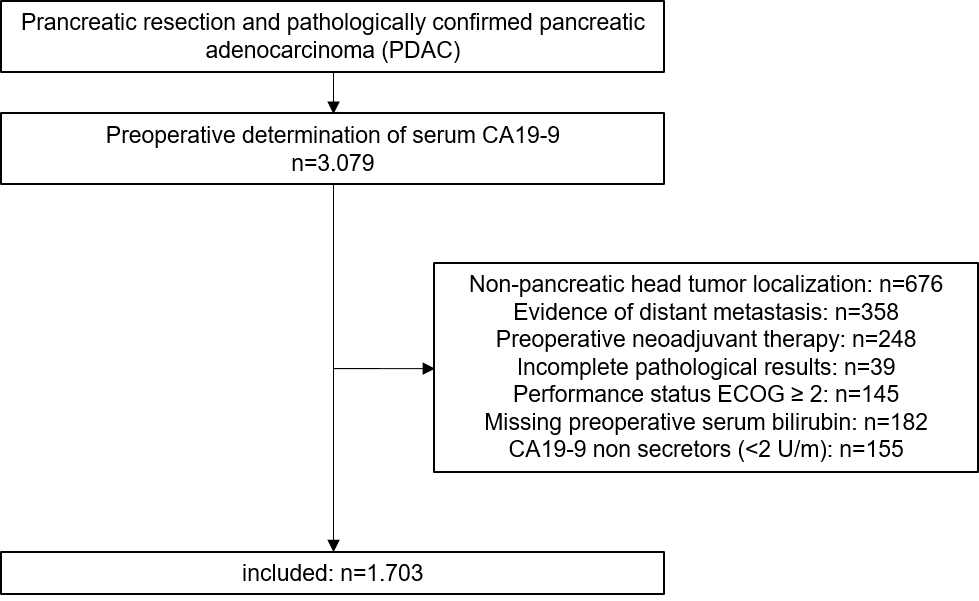
**

Flow chart with in- and exclusion criteria of the study population. ECOG = Eastern Cooperative Onclogy Group; CA19-9 = carbohydrate antigen 19-9

**Supplementary Figure 2**


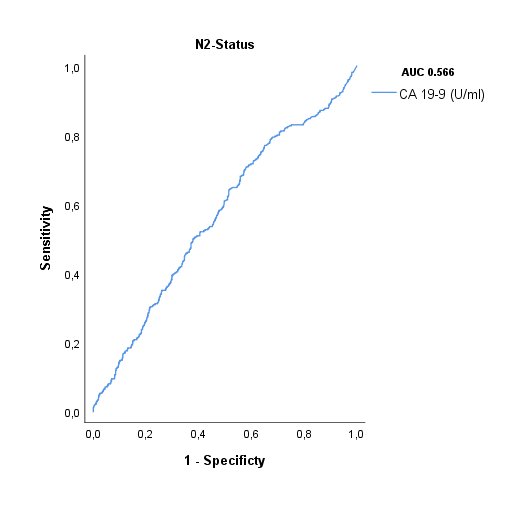

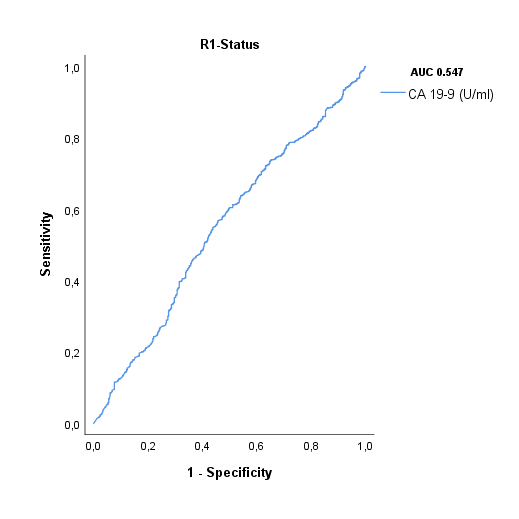


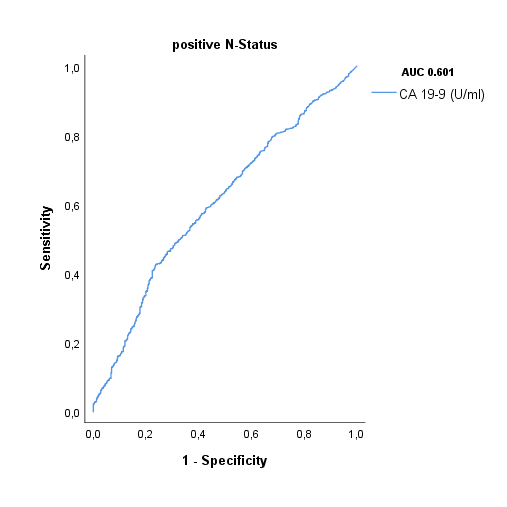


Receiver Operating Characteristic (ROC) curve analysis to define the optimal cut-off point for CA19-9 (Carbohydrate antigen 19-9), accuracy in prediction of R1 resection status, N2-status or nodal positivity in the entire patient cohort.

**Supplementary Figure 3**

*
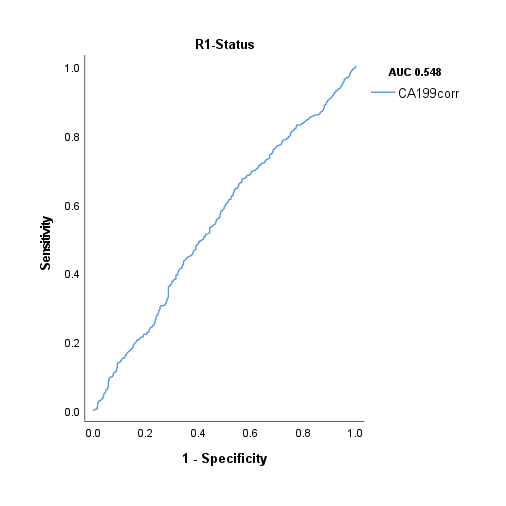

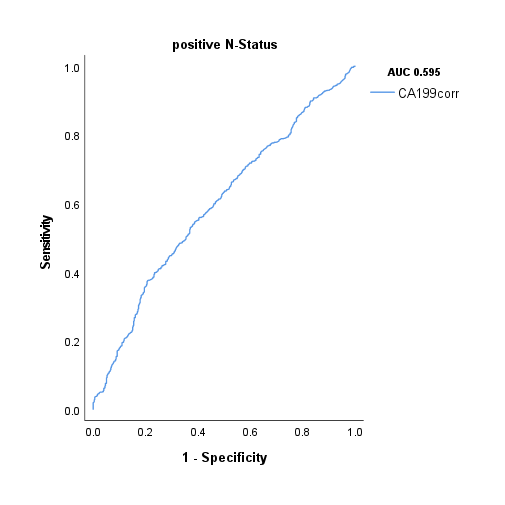
*

*
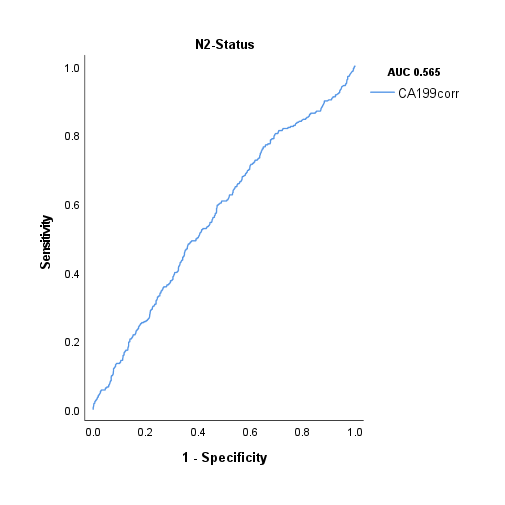
*

Receiver Operating Characteristic (ROC) curve analysis to define the optimal cut-off point for CA19-9 (Carbohydrate antigen 19-9) after correction by concurrent hyperbilirubinemia, accuracy in prediction of R1 resection status, N2-status or nodal positivity in the entire patient cohort.
